# Pain first: rethinking early analgesia in emergency trauma care

**DOI:** 10.1186/s13049-026-01571-y

**Published:** 2026-02-12

**Authors:** Luca Carenzo, Marius Rehn, Martin W. Dünser

**Affiliations:** 1 Department of Anesthesia and Intensive Care Medicine, IRCCS Humanitas Research Hospital, Rozzano, Milano Italy; 2 Air Ambulance Department. Division of Prehospital Services, Oslo University Hospital, Oslo, Norway; 3 Institute of Clinical Medicine, University of Oslo, Oslo, Norway; 4 Department of Research and Development, Norwegian Air Ambulance Foundation, Oslo, Norway; 5 Department of Anesthesiology and Critical Care Medicine, Kepler University Hospital and Johannes Kepler University, Linz, Austria; 6 OEAMTC Flugrettung, Vienna, Austria

Despite being a well-known issue, oligoanalgesia continues to be widely prevalent in emergency care. Observational studies indicate that acute pain is often undertreated, even in controlled healthcare settings [1]. This issue is usually discussed in terms of *process,* such as whether pain relief was provided, which drug was used, or how quickly it was administered, rather than as a patient-centred outcome: whether the patient experienced meaningful relief of pain and distress, and any side effects.

From the patient’s perspective, what matters is not whether an opioid was administered but whether their pain was reduced to a manageable level. It also matters if anxiety and distress were relieved and if treatment facilitated handling, cooperation, and feeling safe. Untreated or inadequately controlled acute pain is not harmless. Beyond immediate suffering, inadequate early pain relief can impede cooperation during rescue and transport, increase stress responses, and adversely affect the patient's memory of care.

In recent articles published in the *Scandinavian Journal of Trauma, Resuscitation and Emergency Medicine*, two studies examine early prehospital pain management in trauma patients using non-intravenous approaches. In the study by Pietsch et al*.* [2], oral transmucosal fentanyl citrate (OTFC) was evaluated with particular attention to age- and sex-related differences, whereas in the studies by Simensen et al*.* [3, 4], inhaled methoxyflurane was examined as an alternative strategy for early trauma analgesia. Together, these studies highlight how effective pain relief can be initiated in trauma patients early during the prehospital phase, including by trained non-physician providers.

The results of both studies have important implications. With appropriate governance and training, effective pain relief can be initiated at the earliest point of patient contact, well before physician-led teams arrive. While the feasibility of applying this model will depend on local laws, scope-of-practice regulations, and medical oversight, it sends a clear message: effective pain management does not need to wait for advanced care to become available.

Together, these two studies build on an established body of evidence supporting early, non-intravenous analgesia in the prehospital setting and address complementary aspects of its clinical relevance. By examining age- and sex-related differences in analgesic response and feasibility across delivery models, Pietsch et al. collectively address an important equity issue, highlighting that effective pain relief should not vary with demographic characteristics or provider assumptions. The absence of clinically meaningful differences in effectiveness or adverse events across patient groups supports the principle that early pain management should be universal rather than selective, guided by patient experience rather than age, sex, or perceived vulnerability.

As with all observational research, these findings warrant cautious interpretation. Such designs are inherently susceptible to selection bias, incomplete documentation, and confounding by indication. Pain scores, while patient-reported, may be inconsistently recorded, and adverse events may be underreported. At the same time, the role of non-intravenous analgesia is increasingly being evaluated in comparative clinical trials rather than observational studies alone. Recent randomised evidence comparing non-intravenous and intravenous strategies in prehospital care suggests that non-intravenous approaches can provide effective early pain relief while avoiding delays related to vascular access [4]. However, analgesic effectiveness alone does not capture the full spectrum of patient-centred outcomes. Functional impact, patient experience, cost and resource use, and trade-offs between pain relief and side effects remain poorly characterised in the prehospital setting.

## Analgesia must start on scene

A common barrier to effective prehospital pain management is the belief that analgesia can be delayed until there is full access to the patient, extrication, monitoring, or physical examination is completed. This sequence reflects a clinician-centred rather than a patient-centred perspective. Pain management should instead be addressed immediately at the scene, along with other life-saving interventions as needed. Early pain relief does not require a complete diagnosis; it only requires recognizing that pain is a valid clinical problem. Interventions that can be delivered rapidly, safely, and without delaying transport are therefore particularly valuable.

## Analgesia without intravenous access: a key advantage

A shared and defining feature of both studies is the use of analgesic strategies that do not require intravenous access. This enables early, safe initiation of effective pain relief across a broader range of prehospital contexts, without prolonging scene time, with important implications for multiple civilian care settings Importantly, this approach does not preclude the subsequent or concurrent use of intravenous or multimodal analgesia, which remains guided by clinical and logistical considerations; in many settings, non-intravenous analgesia may serve as a bridge to intravenous pain management.

### Paediatric populations, frail patients, and difficult-to-obtain IV access

Non-intravenous analgesia is particularly relevant in paediatric populations, where establishing intravenous access can be technically challenging, time-consuming, and itself a significant source of pain and anxiety. By avoiding venous cannulation, transmucosal or inhalational routes may facilitate earlier pain relief, improve patient cooperation, and reduce procedural distress.

### Civilian tactical and ultra-short scene time contexts

In civilian tactical trauma situations, particularly with penetrating injuries in urban settings, scene time is usually kept to under five minutes [5]. In these cases, prolonged on-scene interventions are neither feasible nor desirable. However, pain remains immediate and intense. OTFC analgesia permits initiation of pain treatment without delaying evacuation, allowing patients to receive relief as they board the ambulance or during transport.

### Helicopter emergency medical services and remote environments

Remote environments represent a particularly relevant context for early prehospital analgesia, encompassing both Helicopter Emergency Medical Services (HEMS) and ground-based mountain rescue operations. In HEMS missions, especially for mountaineering or ski-related trauma, rapid evacuation is prioritised, and intravenous access may be unfeasible due to environmental conditions, because of flight constraints, and the risk of nausea or vomiting. In contrast, ground mountain rescue teams often operate in remote areas under challenging conditions. Rescues and evacuations can take a long time, often without immediate access to medical care. In both situations, providing early pain relief is important for obtaining patient cooperation, ensuring safe handling, avoiding hypothermia, and improving rescue efficiency.

### Mass-casualty incidents

Non-IV analgesia also offers strategic benefits for mass-casualty incidents. It provides logistical advantages in scenarios in which many injured people require rapid management with limited resources. Compared to intravenous forms, OTFC is simpler to store, transport, and distribute. This makes it ideal for inclusion in strategic stockpiles [5].

## Expanding the therapeutic reach of prehospital providers

OTFC and Methoxyflurane are additional tools in the prehospital team’s analgesic armamentarium, particularly useful when intravenous access is difficult to obtain or outside of the scope of practice, or when time or resources are limited. Both studies reinforce a fundamental message: effective early pain management in prehospital care is both feasible and safe and should be delivered equitably. Ultimately, the issue is not a lack of pain relief options but the ongoing belief that managing pain is optional or secondary. Recasting pain relief as a patient-centred outcome and enabling its initiation from the first moments of care are crucial steps toward more humane, effective, and patient-centred prehospital care. Future research should aim to strengthen this evidence base through well-designed randomized trials and by specifically addressing understudied populations, including children, in whom early, non-intravenous analgesia may offer particular benefit.

## Data Availability

No datasets were generated or analysed during the current study.
